# Tumor Regression Grading Assessment in Locally Advanced Pancreatic Cancer After Neoadjuvant FOLFIRINOX: Interobserver Agreement and Prognostic Implications

**DOI:** 10.3389/fonc.2020.00064

**Published:** 2020-02-07

**Authors:** Andrea Cacciato Insilla, Caterina Vivaldi, Mirella Giordano, Enrico Vasile, Carla Cappelli, Emanuele Kauffmann, Niccolò Napoli, Alfredo Falcone, Ugo Boggi, Daniela Campani

**Affiliations:** ^1^Department of Surgical, Medical and Molecular Pathology and Critical Care Medicine, University of Pisa, Pisa, Italy; ^2^Department of Translational Research and of New Surgical and Medical Technologies, University of Pisa, Pisa, Italy; ^3^Division of Medical Oncology, Pisa University Hospital, Pisa, Italy; ^4^Diagnostic and Interventional Radiology, Pisa University Hospital, Pisa, Italy; ^5^Department of Transplant and General Surgery, University of Pisa, Pisa, Italy

**Keywords:** tumor regression grade, neoadjuvant therapy, pancreatic cancer, locally advanced, survival

## Abstract

Neoadjuvant therapy represents an increasingly used strategy in pancreatic cancer, and this means that more pancreatic resections need to be evaluated for therapy effect. Several grading systems have been proposed for the histological assessment of tumor regression in pre-treated patients with pancreatic cancer, but issues like practical application, level of agreement and prognostic significance are still debated. To date, a standardized and widely accepted score has not been established yet. In this study, two pathologists with expertise in pancreatic cancer used 4 of the most frequently reported systems (College of American Pathologists, Evans, MD Anderson, and Hartman) to evaluate tumor regression in 29 locally advanced pancreatic cancers previously treated with modified FOLFIRINOX regimen, to establish the level of agreement between pathologists and to determine their potential prognostic value. Cases were additionally evaluated with a fifth grading system inspired to the Dworak score, normally used for colo-rectal cancer, to identify an alternative, relevant option. Results obtained for current grading systems showed different levels of agreement, and they often proved to be very subjective and inaccurate. In addition, no significant correlation was observed with survival. Interestingly, Dworak score showed a higher degree of concordance and a significant correlation with overall survival in individual assessments. These data reflect the need to re-evaluate grading systems for pancreatic cancer to establish a more reproducible and clinically relevant score.

## Introduction

Pancreatic cancer (PC) is the third leading cause of cancer-related death in the United States, with 45,750 projected deaths in 2019 and it is expected to become the second leading cause within few years (1). Mortality rates are almost as high as incidence rates, and from 2014 to 2018 the 5-year survival rate increased only from 6 to 9% (2). PCs are clinically classified as resectable, borderline resectable, locally advanced, and metastatic according to the degree of involvement of collateral blood vessels or presence of metastasis. Although surgery is generally considered the only curative strategy for PC, only a minority of patients, usually reported between 10 and 20%, present at diagnosis with resectable disease. Upfront treatment with chemotherapy with or without radiation therapy and subsequent evaluation for surgery represents an increasingly used strategy in borderline resectable (BR) and locally advanced pancreatic cancer (LAPC) and has shown interesting results even in resectable disease (3–5). In particular, even in the absence of a clear survival benefit upfront medical treatment in LAPC seems to add a significant benefit in terms of downstaging of primary carcinoma, reduction of the risk of positive margins (R1) after histological evaluation, and of local recurrence (6–8). However, standard therapeutic strategies for the treatment of LAPC have not been established yet, and many studies have been published reporting various protocols based on combinations of different chemotherapeutic agents (5). The combination of 5-fluorouracil, oxaliplatin, and irinotecan (FOLFIRINOX) has been associated with notable results in terms of response rate, conversion to surgery and overall survival in different case series (9).

According to the College of American Pathologists (CAP), tumor response in pre-treated patients with PC should be reported. However, to date, a standardized and widely accepted grading system for the histological evaluation of tumor regression grade (TRG) in PC has not been established. The CAP protocol itself states that other systems for the assessment of TRG in PC can be alternatively used (10). In general, current TRG systems for PC are based on a semiquantitative evaluation of criteria like the destruction of viable cancer cells or the extent of fibrosis induced by treatment. To our knowledge, only one study evaluated reproducibility and degree of concordance among pathologists for these systems, reporting a high level of interobserver variability and absence of easily reproducible criteria (11). Moreover, the significance of pathologic response to pre-operative therapy in PC is not well defined, and while some studies reported the clinical relevance of complete or almost complete response after treatment, little is known for the majority of patients who present with partial response (12–15). In this study, two pathologists with expertise in PC used 4 of the most frequently reported systems, CAP, Evans, MD Anderson (MDA), and Hartman, to evaluate tumor regression in 29 LAPC, to critically report flaws and limits of current scores and establish the level of agreement between pathologists. All Scores were also correlated to clinical follow up to determine their potential prognostic value. Cases were additionally evaluated with a fifth grading system inspired to the Dworak score (iDworak), normally used for colo-rectal cancer (16). The concept for an additional grading system resulted as an attempt to identify an alternative, easier and clinically relevant option, not based on previously mentioned criteria.

## Materials and Methods

### Patients and Tumor Specimens

Our study group consisted of 29 patients who underwent surgical resection after neoadjuvant chemotherapy for LAPC at the General Surgery and Transplantation Unit of the University Hospital of Pisa (Italy) between 2011 and 2018. Tumor bed, collateral parenchyma, and peripancreatic tissues were completely sampled and embedded according to our institutional protocol for pancreatic surgery after neoadjuvant therapy. Formalin-fixed and paraffin-embedded samples from all the 29 patients were sliced with a microtome, and routine hematoxylin and eosin staining was performed.

### Therapeutic Protocol

Patients with cytologically confirmed LAPC (cT4, cN0-2, cM0) considered unresectable according to definition of the NCCN guidelines v. 03.2019 (17), with ECOG Performance Status 0–1, aged 18–75 years, were treated with modified FOLFIRINOX (mFOLFIRINOX) (irinotecan 165 mg/sqm, oxaliplatin 85 mg/sqm, folinate 200 mg/sqm, 5-fluorouracil 3,200 mg/sqm in 48 h) every 2 weeks (18). Minimum number of cycles was 4 and all patients were re-evaluated for surgery every 4 cycles by a multidisciplinary team, according to protocol in our institution. Patients usually continued chemotherapy until surgery became feasible, up to a maximum of 12 cycles Tumor assessment was performed by computed tomography (CT) scan of abdomen and chest every 8 weeks and multidisciplinary team evaluated patients after every CT scan. Objective responses were evaluated according to RECIST criteria v.1.1 (19). Upon completion of neoadjuvant chemotherapy patients were still considered to have an unresecatbale tumor if distant metastasis were detected, if involved vessels could not be safely reconstructed, and if operative risk was deemed to high or was not accepted by the patient. Absence of radiologic downstaging was not seen as a contraindication to resection. Before proceeding with open surgery, all patients were explored laparoscopically to rule out occult metastatic disease. Only patients who underwent definitive surgery with curative intent were included in this analysis. Data regarding baseline characteristics, treatment and follow up were retrospectively collected.

### TRG Evaluations

**Figure 1:**
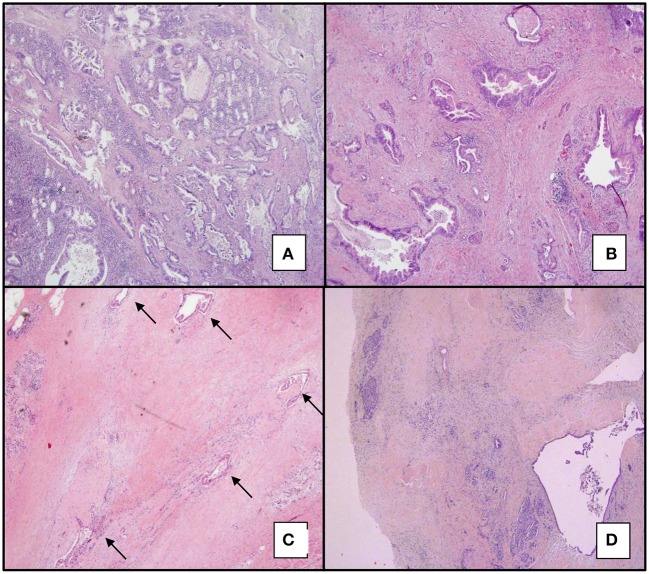
iDworak TRG system applied to PCs. iDworak TRG system applied to PCs. **(A)** (4x): dominant tumor mass with poor fibrosis. (Grade 0). **(B)** (4x): dominant tumor mass with obvious fibrosis (Grade 1). **(C)** (4x): few tumor cells or groups (easy to find, Grade 2); arrows indicate neoplastic glands. **(D)** (4x and 20x): very few tumor cells (difficult to find microscopically, Grade 3); in this case neoplastic cells were detected in only one slide. Total response (corresponding to score 4) was not present in our group of patients.

All cases were retrospectively reviewed by two pathologists with expertise in pancreatic malignancies and TRG was assessed for all specimens, according to four of the most commonly used grading systems for PC: CAP, Evans, MDA, and Hartman (Table 1). Ratio between stroma and epithelial component of the tumor was also assessed with CAP score and results were compared to the number of chemotherapeutic cycles, in order to define some possible correlations. All cases were additionally evaluated with a fifth grading system inspired to the Dworak score (iDworak), normally used for colo-rectal cancer. According to this latter score, PCs were evaluated as follows: grade 0 for dominant tumor mass with poor fibrosis; grade 1 for dominant tumor mass with obvious fibrosis; grade 2 for dominant fibrotic tissue with few neoplastic cells/glands, easy to find; grade 3 for dominant fibrotic tissue with very few neoplastic cells/glands, difficult to find; grade 4 for complete regression (Figure 1). In particular, grade 0 was assessed as tumors with dominating glandular component and only minimal, baseline fibrosis around neoplastic glands; grade 1 corresponded to tumors with larger fibrotic areas among neoplastic glands, but still with a predominant glandular component; in grade 2, “easy to find” was considered as few neoplastic glands/nests easily detectable at low magnification; in grade 3 “difficult to find” was considered as rare neoplastic cells/small glands, not detectable at low magnification, that required a more meticulous and thorough examination. Only the epithelial component of the tumor was evaluated to assess the iDworak score, while cytopathic effect was not taken into consideration. All scores were assigned after the complete evaluation of all slides and not based on representative tumor samples. TRG evaluations were performed independently by each pathologist, and the level of concordance was statistically established; a discussion after comparison of the scores was carried out and discordant cases were re-evaluated at a multi-ocular microscope.

**Table 1:** Regression grading systems applied in the study.

***College of American Pathologist (CAP)* (10)**
** • *Grade 0*:** no viable cancer cells (complete response) ** • *Grade 1*:** single cells or rare small groups of cancer cells (near complete response) ** • *Grade 2*:** residual cancer with evident tumor regression, but more than single cells or rare small groups of cancer cells (partial response) ** • *Grade 3*:** extensive residual cancer with no evident tumor regression (poor or no response)
***MD Anderson* (12)**
** • *Grade 0*:** no residual carcinoma ** • Grade 1:** patients with minimal residual carcinoma (single cells or small groups of cancer cells, <5% residual carcinoma) ** • *Grade 2*:** patients with 5% or more residual carcinoma
***Evans* (20)**
** • *Grade I*:** characteristic cytologic changes of malignancy are present, but little (<10%) or no tumor cell destruction is evident. ** • *Grade II:*** in addition to characteristic cytologic changes of malignancy, 10-90% of tumor cells are destroyed ** ▪ *IIa*:** destruction of 10-50% of tumor cells ** ▪ *IIb:*** destruction of 51-90% of tumor cells ** • *Grade III:*** few (<10%) viable-appearing tumor cells are present ** ▪ *IIIM:*** sizable pools of mucin are present ** • *Grade IV:*** no viable tumor cells are present ** ▪ *IVM:*** acellular pools of mucin are present
***Hartman* (21)**
** • *Marked Response:*** no residual tumor or rare, single cancer cells or small groups of cancer cells (glands) with marked cytopathic effect present within a fibrotic stroma ** • *Minimal to Moderate Response*:** Residual tumor present; includes small groups of cells/glands without evidence of cytopathic effect, cells/ glands outside the main fibrotic mass, and/or 0.5% of the main fibrotic mass with cancer/glands, with or without cytopathic effect ** • *Poor Response:*** No definite evidence of treatment effect; extensive (90%) residual cancer; only minimal cytopathic effect, and baseline fibrosis is present
***iDworak* (16)**
** • *Grade 0:*** dominant tumor mass with poor fibrosis ** • *Grade 1:*** dominant tumor mass with obvious fibrosis ** • *Grade 2:*** dominant fibrotic tissue with few neoplastic cells/glands, easy to find ** • *Grade 3:*** dominant fibrotic tissue with very few neoplastic cells/glands, difficult to find ** • *Grade 4:*** complete regression

### Statistical Analysis

Statistical analysis was performed using the Kendall coefficient of concordance (k) to assess the level of agreement between pathologists. Results with this test can range from −1, corresponding to complete disagreement to +1 for total agreement; 0 indicates the absence of correlation. All TRG scores were correlated with Overall Survival (OS), OS from Surgery (OSS), Progression-Free Survival (PFS) and Disease-Free Survival (DFS). OS was defined as the time from the first day of chemotherapy until the day of death from any cause, PFS was defined as the time from the first day of chemotherapy until the day of disease progression or death from any cause. Post-surgical OS (OSS) was defined as the time from surgery until the day of death, DFS was defined as the time from surgery until the day of disease progression or death from any cause. Patients alive at the time of analyses were censored at the date of their last follow-up visit, whereas those without disease progression were censored at the time of the last radiologic assessment. Survival analyses were performed using the Kaplan-Meyer method and differences in survival were compared using the log-rank test, setting statistical significance at *p* < 0.05 for a two-sided test. Statistical and survival analyses were performed using IBM SPSS statistic software, version 21.

## Results

### Clinical-Pathological Features

A group of 29 patients with LAPC was retrospectively selected for this study. Main baseline characteristics are reported in Table 2.

**Table 2:** Baseline characteristics of 29 patients with PC.

**Characteristic**	***N* = 29**	**%**
**Gender**		
Male	16	55.2
Female	13	44.8
**ECOG performance status**		
0	24	82.8
1	5	17.2
**Age**		
Median	58	
Range	(34–74)	
**Tumor location**		
Head	23	79.4
Body-Tail	6	20.6
**Baseline vessel involvement**		
Superior mesenteric artery	22	75.8
Celiac axis	4	13.8
Hepatic artery	8	27.6
Portal vein	10	34.5
Superior mesenteric vein	21	72.4
**Baseline Ca19.9 level**		
Median	91	
Range	(3–3924)	

### Clinical and Pathological Outcome

A median number of induction-mFOLFIRINOX cycles was 8 (range 3–12). One patient underwent surgery after 3 cycles due to hematologic toxicity. Radiological response rate according to RECIST 1.1 criteria was 55.2% (16 patients) and disease control rate was 100% (22). Fifteen patients (53.4%) underwent total pancreatectomy, 11 (36.6%) pancreatoduodenectomy (PD), and 3 (10%) distal pancreatectomy. In 26 cases out of 29, vascular resection was performed: 17 patients underwent both the superior mesenteric vein (SMV) and artery (SMA) resection, while 9 patients underwent only SMV resection. After histological evaluation of surgical specimens, 22 cases were diagnosed as ductal adenocarcinoma (PDAC), 5 as adenocarcinoma derived from intraductal pancreatic mucinous neoplasia (IPMN), and 1 as adenosquamous carcinoma; in 1 case, cancer histotype was not assessed due to the small number of neoplastic cells left after neoadjuvant treatment. Gross dimension of the tumor, when assessable, ranged from 1.2 to 6 cm. In 27 cases tumor extended to peripancreatic tissue; in addition, in 3 cases infiltration of both SMV and SMA was reported, 7 cases presented with only SMV infiltration, while in 3 cases only SMA invasion was present. Lymph node metastasis were described in 22 patients (11 N1 and 11 N2). All histological evaluations were reviewed according to the 8th AJCC staging system (23).

### TRG Evaluation and Agreement

None of the 29 cases examined presented with complete regression after microscopic examination. Concordance between pathologists resulted statistically significant for every score used, however, different levels of agreement were observed (Table 3). The highest degree of concordance (0.913 and 0.830) was reached with iDworak and Hartman grading systems, while Evans and MDA obtained the lowest one (respectively, 0.566 and 0.521). CAP grading system revealed an intermediate degree of concordance (0.644).

**Table 3:** Level of concordance between pathologists.

**Grading System**	**K**	***p***
CAP	0.644	< 0.05
MD Anderson	0.521	< 0.05
EVANS	0.566	< 0.05
HARTMAN	0.830	< 0.05
DWORAK	0.913	< 0.05

### Evans

Evans grading system revealed the lowest degree of concordance between pathologists, with total consensus in 9 patients. Grade II was the most frequently used, assessed, respectively, in 17 and 19 cases; in particular, grade IIa was reported 12 and 13 times, while grade IIb in 5 and 6. All the 9 cases with total consensus were scored as grade IIa. Disagreement about grade III, corresponding to <10% of viable-appearing neoplastic cells, was the most frequently observed, respectively, 5 and 2.

### MDA

MD Anderson revealed the highest level of consensus among all the four conventional grading systems, with a total agreement reached in 26 cases; however, the k coefficient obtained was 0.521. MDA represented the only two-tiered grading system in this study, also considering the absence of total response (grade 0), so all disagreements encountered were between grade 1 and 2.

### CAP

Total agreement was observed in 21 cases. Grade 2 was the score most frequently employed, respectively, 13 and 17 times, with consensus reached in 11 cases. Poor or no response (grade 3) was reported in 12 and 10 cases, while grade 1, near complete response, in 4 and 2. Disagreement between grade 1 and 2 was the most recurrent. CAP score was also used to compare total number of chemotherapeutic cycles with the amount of fibrosis detected in tumor samples, without any significant correlation (*p*: 0.77).

### Hartman

Hartman score revealed a good level of agreement, with consensus in 25 cases. Poor response was the score assigned most frequently (16 times) with total agreement in 15 out of 16. Marked response was assessed in 2 cases by both pathologists but with consensus in only 1 patient.

### iDworak

The largest consensus (27 cases) was obtained with iDworak score. Grade 1 was the most frequently used by both pathologists, 17 cases out of 29. Concordance was not reached in 2 cases, with disagreement between grade 0/1 in 1 case and between 1/2 in the other one. Grade 3 was assessed in only 1 case by both pathologists and for the same patient. Grade 0 was used respectively in 3 and 4 cases.

### TRG and Follow Up

At a median follow up of 28.9 months 22 patients experienced documented disease progression and 20 died (1 death was not related to disease progression). Median PFS from starting of chemotherapy was 13.8 months and median OS was 20.8 months, while DFS and OSS were 8.0 and 13.8 months, respectively. Tumor regression after neoadjuvant chemotherapy according to all scores was not associated with improved survival outcome (*p* > 0.05) except for iDworak. Complete results are reported in Table 4.

**Table 4:** #P1: Pathologist 1; #P2: Pathologist 2.

**Grading system**	**OS**	***p***	**OSS**	***p***	**PFS**	***p***	**DFS**	***p***
	**mOS (months)**		**mOSS (months)**		**mPFS (months)**		**mDFS (months)**	
CAP 1 vs. 2 vs. 3 #P1 #P2	17.6 vs. 23.2 vs. 14.5 11.6 vs. 23.7 vs. 13.4	0.958 0.688	11.7 vs. 14.5 vs. 10.1 8.2 vs. 14.5 vs. 8.8	0.942 0.639	13.6 vs. 17 vs. 13.4 11 vs. 17 vs. 12.5	0.788 0.194	7.7 vs. 11.9 vs. 8 7.5 vs. 11.9 vs. 5.8	0.947 0.512
MDA 1 vs. 2 #P1 #P2	17.6 vs. 23.2 NR vs. 20.8	0.782 0.203	11.7 vs13.8 NR vs. 12.5	0.752 0.179	13.6 vs. 16 NR vs. 15.5	0.838 0.185	7.7 vs. 9.5 NR vs. 9.3	0.741 0.204
EVANS 1 vs. 2 vs. 3 vs. 4 #P1 #P2	14.5 vs. 25.1 vs. 23.7 vs.24.6 14.5 vs. 24.6 vs. 23.2 vs. 11.6	0.357 0.633	10.1 vs. 12 vs. 18.6 vs. 11.7 9.5 vs. 14.5 vs. 16.7 vs. 8.2	0.428 0.715	11 vs. 17.2 vs. NR vs. 13.6 13.4 vs. 17.2 vs. 13.6 vs. 11	0.315 0.535	5.3 vs. 9.5 vs. 11.9 vs. 7.7 5.8 vs. 11.9 vs. 7.7 vs. 7.5	0.48 0.828
HARTMAN 0 vs. 1 vs. 2 #P1 #P2	20.8 vs. 23.2 vs. 11.6 19.8 vs. 20.8 vs. 11.6	0.402 0.860	14 vs. 11.7 vs. 8.2 13.8 vs. 12.5 vs. 8.2	0.207 0.677	16 vs. 13.6 vs. 11 16 vs. 13.6 vs. 11	0.747 0.568	9.5 vs. 7.7 vs. 7.5 9.5 vs. 7.7 vs. 7.5	0.816 0.213
iDWORAK 0 vs. 1 vs. 2 vs. 3 vs. 4 #P1 #P2	14.5 vs. 19.8 vs. NR vs. 11.6 14.5 vs. 20.8 vs. 25.9 vs. 11.6	0.016 0.025	8.8 vs. 14 vs. 12.5 vs. 8.2 8.8 vs. 14 vs. 12.5 vs. 8.2	0.091 0.141	11 vs. 13.4 vs. 25.9 vs. 11 11 vs. 15.5 vs. 25.9 vs. 11	0.023 0.134	5.3 vs. 8 vs. 12.5 vs. 7.5 5.3 vs. 9.3 vs. 12.5 vs. 7.5	0.212 0.457

**Figure 2:**
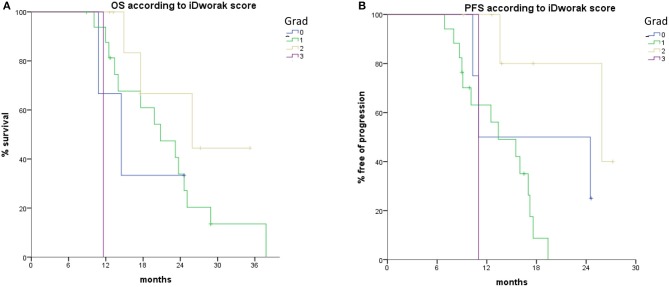
Histopathologic treatment effect according to iDworak score is associated with improved **(A)** OS and **(B)** PFS. PFS, progression free survival; OS, overall survival.

iDworak was significantly correlated to OS in individual evaluations of both pathologists (*p* = 0.016 and *p* = 0.025, respectively); it correlated with PFS only in one individual evaluation (*p* = 0.023 and *p* = 0.134, respectively), had a trend toward increased OSS only in one individual evaluation (*p* = 0.091 and 0.141) while did not show any stratification of the different groups in terms of DFS in either case (*p* = 0.212 and 0.457, respectively) (Figure 2).

## Discussion

The histological assessment of tumor regression in pancreatic cancer after neoadjuvant therapy is not a standardized procedure. Different grading systems are currently in use but criteria like reproducibility, degree of agreement, and clinical correlation have not been validated for many of them (10, 12, 20, 21, 24–26). In this study, two pathologists with expertise in PC (A.C.I. and D.C) decided to use 4 of the most frequently reported systems (CAP, Evans, MDA, and Hartman) to evaluate tumor regression in 29 LAPC, to establish the level of concordance. Scores obtained were also correlated to clinical follow up to determine their potential prognostic value. All cases were additionally evaluated with a fifth grading system inspired to the Dworak score (iDworak), normally used for colo-rectal cancer. The current TRG systems are mainly based on a semiquantitative evaluation of the extent of fibrosis induced by therapy or on the destruction of viable neoplastic cells, but these criteria are purely subjective and prone to a high risk of interobserver variability. In addition, limited data suggest the usefulness of these grading systems as a prognostic index, and clinicians usually do not consider them for therapeutic decisions (8, 11, 13, 14, 21, 27). For these reasons, the concept for an additional grading system resulted as an attempt to identify an alternative, easier and clinically relevant option, not based on previously mentioned criteria. To our knowledge, only one study has been previously published by Kalimuthu et al. about concordance and interobserver variability on TRG systems in PC (11). In our study, different levels of agreement between pathologists were observed, but concordance resulted statistically significant for every score used. Evans revealed by far the lowest level of consensus, with agreement in just 9 cases out of 29 and k coefficient of 0.566. Evans is a 4-tiered score based on the percentage of residual viable neoplastic cells in the tumor after therapy. In his paper, Evans described the morphological aspects of viable and non-viable neoplastic cells (20); however, these aspects can be also frequently identified in non-treated cancers or differently they may not be present, making grading systems that require an assessment of viability challenging and quite questionable. Moreover, the original tumor size should be known to report a realistic percentage of the residual neoplastic cell, but usually this cannot be reliably obtained even with modern imaging modalities; consequently, even grading systems that assess residual tumor with percentage cut-offs are often arbitrary and approximative (8, 28). In this study, disagreement on Evans grade III was the most frequently observed and resulted from a different assessment of therapy effects and residual amount of tumor performed by pathologists. A better consensus was obtained for grade I, corresponding to little or no tumor cell destruction. During a post-study discussion, pathologists agreed on the difficulty and tendency to a personal evaluation of cell viability; they also stressed the fact that tumor regression is usually not uniform throughout the lesion and sometimes can be difficult to perform a global score based on all slides. In particular, the presence of areas with a very different response to therapy can increase the interobserver variability, with the risk of over or underestimate cases with scores close to cut-offs.

MDA showed the highest level of consensus among the four conventional grading systems, with agreement in 26 out of 29 cases, but with a k coefficient of 0.521. Interestingly, also in the aforementioned study by Kalimuthu et al. MDA demonstrated the highest level of consensus among pathologists but with a k score ranging from 0.00 to 0.67 (11). MDA is a 3-tiered score proposed by Chatterjee et al. as a modification of CAP grading system, and based on the percentage of residual tumor, with a 5% cut-off value to distinguish cases with a marked response from those with moderate to poor response (12). Chatterjee reported that patients with grade 0 and 1 have significant better survivals than patients with grade 2, and these data were subsequently validated in a second study by Lee et al. (13). More studies also reported that complete or almost complete response in PC usually correlates with better survivals (15, 29–33). However, these patients are uncommon in daily practice (30, 32): in the aforementioned studies by Chatterjee and Lee cases scored as grade 0 and 1 represented just the 16% of all patients evaluated. It is also necessary to stress that patients with moderate to poor response often present with different survivals, and so they should not be included in the same prognostic group (5, 34). In our cohort, for instance, patients classified as grade 2 with the MDA score presented a median OS of 19.3 months, with a variable range from 8.9 to 37.8 months. Hence, MDA appears as an over-simplified grading system able to give some relevant information in a minority of cases, but unable to provide a realistic prognostic stratification that is frequently observed in the majority of patients with PC. It is also noteworthy that *Chatterjee* was the only pathologist to review all the slides in his study, to exclude the interobserver bias; instead, 3 different pathologists performed all the evaluations in the second study, but no details were reported about interobserver variability.

CAP is a 4-tiered grading system that evaluates the level of response by the ratio between residual tumor and fibrosis. In this study, a total agreement was observed in 21 cases, with an intermediate k of 0.644; grade 2, which means partial response, was the score most frequently employed. Disagreement between grade 1 and 2 was the most recurrent; as a matter of fact, distinction between “single cells/rare small groups of cancer cells,” as reported for grade 1, and “residual tumor with evident regression,” for grade 2, can be extremely subjective, especially in those cases with good response. In post study discussion, pathologists debated about the concept of “small groups of cancer cells,” considering that is not clear if the presence of a small number of complete neoplastic glands on few slides could be enough to assess grade 2. Another important issue lies in the difficulty of distinguishing fibrosis due to regression. Pancreatic cancer is typically characterized by a variable amount of desmoplastic stroma, so evaluation of therapy effect by the degree of fibrosis is extremely challenging (14). To date, no guidelines have been reported to distinguish desmoplastic reaction from therapy-induced fibrosis. Some studies attempted to analyze the molecular composition of pancreatic stroma after treatment, to identify markers relevant to distinguish therapy-induced from tumor-associated fibrosis, but still without success (35). In this study, CAP score was also used to compare the total number of therapeutic cycles and the amount of fibrosis detected in tumor samples, without any significant correlation; however, the aforementioned difficulty in separating desmoplastic reaction from post-therapeutic fibrosis may suggest the poor reliability of this result. The presence of some features such as foamy macrophages, multinucleated giant cells or hemosiderin deposition have also been reported as potential regression markers due to therapy effect; however, pancreatic cancer is frequently associated to coexisting pancreatitis, often due to obstructive effects, that may occur with comparable histological features. Considering this burden, pathologists involved in this study agreed that regression should be evaluated only taking into account residual tumor, without fibrosis.

Hartman is the last current grading system used in this study. After a review of the literature, Hartman appears as the least frequently used score to assess tumor regression grade in pancreatic cancer and it was not taken into consideration by Kalimuthu et al. (11). Interestingly, in this study, we observed a very good consensus with this score, 25 cases out of 29, and the k coefficient obtained (0.830) was higher than Evans, MDA, and CAP. In his paper, Hartman proposed a 3-tiered modification of CAP grading system, as performed for the MDA, with some integration from other scores like Ishikawa and Evans (21). The idea was to provide a simple and practical approach to grade treatment response, not based on the assessment of viability, and able to reduce intra and interobserver variability. However, some discrepancies can be observed. Despite the intention of the author, this score requires the evaluation of cytopathic effects due to therapy, and percentage cut-offs are also necessary to report residual tumor, not solving the issue of personal assessments. Despite all this, we observed a good agreement between pathologists, but at the same time, this score was associated with the highest number of cases classified as poor response. The difficulty in comparing grading systems with different tiers could explain these results; in addition, the presence of many combined, and sometimes time-consuming, criteria for histological assessment can increase the risk of a more inaccurate evaluation. Reproducibility is not the only problem with these grading systems: while TRG has been showed to be significant for the clinical outcomes of various cancers, it is not used to make clinical decisions in pre-treated patients with PC. Even if complete response appears to be clinically significant, data reported by these studies often vary from each other and the absence of a standardized therapeutic protocol and pathological grading systems make it difficult to compare results from the literature (6, 8, 11–13, 30, 32, 36). To reduce variability, a highly selected cohort of patients was considered for this study, with shared radiologic diagnosis of LAPC and all treated with the same therapeutic regimen. All individual histological assessments were compared to OS, OSS, PFS, and DFS but no significant statistical correlation was observed. Interestingly, even MDA score did not reveal any prognostic significance, although the very few cases observed with almost complete response, respectively, 3 and 4, could explain this data. iDworak was the fifth score used in this study. Considering all the previous statements, we tested a different grading system, inspired to a well-known TRG used for colo-rectal cancer, and not based on classic criteria like the extent of fibrosis induced by treatment, cell viability assessment or percentage cut-offs. The amount of residual tumor and also the “easiness” to detect neoplastic cells were the only aspects took into account. iDworak showed by far the highest consensus, 27 cases in 29, and also the best k coefficient (0.913). Interestingly, both pathologists reported fewer cases with “no response” than with other scores, probably due to the lower number of criteria applied that resulted in a less personal and maybe more objective score. Moreover, although the small number of patients selected for this study, iDworak was the only grading system to significantly correlate with OS and it also correlated with OSS and PFS in one individual assessment.

It is important to underline that all the histological assessments reported in this study were performed after the complete submission of the surgical specimen and an overall evaluation of all slides. It is our firm conviction that surgical specimens of pre-treated patients must be entirely sampled and submitted, to perform a reliable evaluation and completely rule out the presence of neoplastic cells. According to our daily experience, particular attention should be paid to peripheral areas where residual tumor can be often detected, especially the duodenal wall, around the main mesenteric vessels and in peripancreatic lymph nodes.

## Conclusions

To our knowledge, this is the second study, after the one proposed by Kalimuthu et al. to assess the level of agreement between pathologists in reporting TRG in pancreatic cancer. Even if concordance resulted statistically significant for every score used, very different levels of agreement were observed, proving that these grading systems are too subjective and inaccurate. All the evaluations were performed by only 2 pathologists and in a small cohort of patients, and this can represent a limit to the study; however, 4 gastro-intestinal pathologists participated to the study by Kalimuthu et al. but still, a poor concordance was reported, even worse than in this study. These results seem to confirm the hypothesis by Kalimuthu that increasing the number of participants is not the solution to improve the level of agreement, especially if criteria used in current scores remains so subjective, personal and sometimes difficult to assess. Furthermore, the evaluations were conducted in a small cohort of patients, but the study population was very selected in terms of stage and preoperative treatment and this is the first report evaluating patients treated with preoperative chemotherapy with FOLFIRINOX regimen without radiotherapy. We also correlated TRG to survivals, and we obtained significant results, beyond the best level of consensus, only with the new proposed score. Even if it was not in the intention of this study to suggest a new TRG system for PC, results obtained with the iDworak highlights that a score mainly based on the evaluation of residual tumor could provide more significant information for a better clinical stratification of patients with PC. Prospective evaluations in larger independent cohorts of patients is needed to validate these results. In addition, further studies about molecular aspects of PC and change in tumor microenvironment during treatment could represent a novel topic to be investigated.

## Data Availability Statement

All datasets generated for this study are included in the article/supplementary material.

## Ethics Statement

Ethical review and approval was not required for the study on human participants in accordance with the local legislation and institutional requirements. Written informed consent for participation was not required for this study in accordance with the national legislation and the institutional requirements.

## Author Contributions

AC and CV conceived the study, carried out data interpretation, and wrote the paper. AC and DC assessed histological evaluations. CV, EV, and CC collected clinical data and performed survival analysis. AF, UB, DC, NN, and EK supervised the findings of this study. MG helped with data collection and analysis.

### Conflict of Interest

The authors declare that the research was conducted in the absence of any commercial or financial relationships that could be construed as a potential conflict of interest.
